# Lane Attribute Classification Based on Fine-Grained Description

**DOI:** 10.3390/s24154800

**Published:** 2024-07-24

**Authors:** Zhonghe He, Pengfei Gong, Hongcheng Ye, Zizheng Gan

**Affiliations:** School of Electrical and Control Engineering, North China University of Technology, Beijing 100144, China; shiwei@mail.ncut.edu.cn (P.G.); yehongcheng@mail.ncut.edu.cn (H.Y.); ganzizheng@mail.ncut.edu.cn (Z.G.)

**Keywords:** lane detection, fine-grained attribute detection, construction of fine-grained attribute data set, attention mechanism

## Abstract

As an indispensable part of the vehicle environment perception task, road traffic marking detection plays a vital role in correctly understanding the current traffic situation. However, the existing traffic marking detection algorithms still have some limitations. Taking lane detection as an example, the current detection methods mainly focus on the location information detection of lane lines, and they only judge the overall attribute of each detected lane line instance, thus lacking more fine-grained dynamic detection of lane line attributes. In order to meet the needs of intelligent vehicles for the dynamic attribute detection of lane lines and more perfect road environment information in urban road environment, this paper constructs a fine-grained attribute detection method for lane lines, which uses pixel-level attribute sequence points to describe the complete attribute distribution of lane lines and then matches the detection results of the lane lines. Realizing the attribute judgment of different segment positions of lane instances is called the fine-grained attribute detection of lane lines (Lane-FGA). In addition, in view of the lack of annotation information in the current open-source lane data set, this paper constructs a lane data set with both lane instance information and fine-grained attribute information by combining manual annotation and intelligent annotation. At the same time, a cyclic iterative attribute inference algorithm is designed to solve the difficult problem of lane attribute labeling in areas without visual cues such as occlusion and damage. In the end, the average accuracy of the proposed algorithm reaches 97% on various types of lane attribute detection.

## 1. Introduction

In recent years, the development of autonomous driving technology has been very rapid. A complete autonomous driving system consists of multiple cores, including environmental awareness, decision planning, and control execution. Among them, environmental perception is the first link of automatic driving, which is located in the key position of vehicle interaction with external environmental information. The main task of environmental perception is to detect and identify vehicles, pedestrians, road traffic signs, marking, and traffic signal control. The focus is on enabling intelligent driving vehicles to better mimic the perception of human drivers so that they can understand their own driving situations and those around them.

In the task of road traffic marking detection, lane detection represents the detection of vehicle driving area to a certain extent, so it has been widely studied. Traditional lane detection methods usually involve preprocessing operations such as denoising and enhancing the original image, and they then use manually designed feature extraction operators to achieve lane detection [1]. This kind of algorithm has low computational complexity and weak robustness, which cannot meet the needs of autonomous driving development. In contrast, the lane detection method based on deep learning is based on a convolutional neural network, which has better robustness, higher accuracy, and has gradually become the mainstream research and application direction. According to different lane line modeling methods, it can be divided into segmentation-based lane line detection algorithm, line anchor-based lane line detection algorithm, line classification-based lane line detection algorithm, key point-based lane line detection algorithm, and function curve fitting-based lane line detection algorithm. Different types of lane detection algorithms have different advantages according to their different modeling methods.

In addition, in the actual urban environment, the attribute categories of lane lines are diverse, including yellow lines, white lines, single lines, and other types. Existing research on lane detection mainly focuses on how to accurately detect the position of lane lines in images in complex scenes. However, in complex urban scenes, in addition to the location information of lane lines, the properties of dotted and solid lines, yellow and white lines, and single and double lines are also crucial to vehicle running. The vehicle’s own sensor can detect the attribute categories of lane lines, especially the attribute changes of different positions of the same lane lines, which can provide decision-making information for tasks such as lateral control and short-distance path planning of autonomous vehicles. This paper calls it fine-grained attribute detection of lane lines.

To sum up, although there has been a lot of research on the location information detection of current lane detection methods, there is relatively little research on the complete detection of the fine-grained attributes of lane lines. This is mainly due to two reasons: one is the lack of open-source lane data set with fine-grained attribute annotation, which makes it difficult to obtain sufficient data support for related research. Second, in the urban environment, lane lines are often affected by no visual cues such as occlusion, which makes it difficult to accurately label fine-grained attributes. Due to this limitation, existing lane attribute classification methods can only judge the overall attribute of each detected lane instance [2,3] or only realize fine-grained lane attribute detection through semantic segmentation, but they cannot distinguish lane instances [4,5].

The main contributions of this paper are as follows:

(1) Aiming at the limitations of previous lane attribute classification methods, this paper designs a lane fine-grained attribute (Lane-FGA) detection method, which includes a lane position detection part and a lane attribute detection part. Firstly, pixel-level attribute sequence points are used to describe the complete attribute distribution of lane lines so as to realize finer granularity attribute judgment of the lane lines. Secondly, based on the idea of row classification, the lane line location information detection algorithm is redesigned to improve the accuracy of the lane line detection method. The experimental results show that this method can detect more complete information of lane lines in complex urban scenes.

(2) Based on ApolloScape traffic marking data set, this paper constructs a lane data set with both lane information and fine-grained attribute information. A cyclic and iterative lane attribute inference method is designed to solve the difficult problem of lane attribute labeling in areas without visual cues such as occlusion.

## 2. Related Work

### 2.1. Lane Position Information Detection

The lane detection algorithm based on segmentation regards different lane lines in image information as different categories of objects, and it realizes lane detection through semantic segmentation. Based on this, Pan et al. [6] regarded lane recognition and detection as a multiclass semantic segmentation task, and they designed a new convolutional module according to the prior characteristics of the long and thin shape of the lane, thus effectively improving the detection ability of objects with long and thin shape characteristics. Neven et al. [7] proposed a detection algorithm that could predict the indefinite number of lane lines. After the feature extraction network, two detectors were connected in parallel, and one divided the foreground and background pixels of the lane lines in the image using the semantic segmentation method. Another was used to calculate the high-dimensional embedding coordinates of the lane line pixels, and then they used a clustering algorithm to distinguish the foreground pixels of the lane lines according to the high-dimensional coordinates obtained using position embedding. Considering that the current lane detection algorithm mainly focuses on a single image (frame) and ignores the dynamic information in the video, Zhang et al. [8] therefore proposed a method of lane detection using video sequences to enhance the feature representation of the current frame by concentrating the local and global memory features of other frames, thus improving the accuracy rate of the lane detection algorithm based on segmentation. A large data set of lane detection based on video sequence has also been open sourced. Inspired by the simple geometric structure of lanes, Lee et al. [9] proposed a self-attention method for lane detection, called the Extended Self-attention (ESA) module, which improves the anti-interference ability of the lane detection model by predicting the confidence of the vertical and horizontal lane lines in the image.

The target detection task is one of the basic tasks in the field of computer vision. In the target detection task, a large number of prior anchor frames with different sizes and proportions are usually preset on the single or multilayer feature map, and the target object is detected by regression through the prior anchor frames. Comparing with the target detection algorithm based on the anchor frame, Li et al. [10] proposed a lane detection method based on the anchor, which introduced the idea of the anchor frame into the lane detection task to improve the accuracy of lane detection. The line anchor is defined as a ray extending from the edge of the image matrix. A certain number of anchor points are set on the left and right of the image matrix, and each anchor point extends a number of rays from different angles as a prior line anchor. Tabelini et al. [11] integrated the attention mechanism with line anchors and generated an attention matrix by using the interaction between the regression predicted values of different prior line anchors so as to achieve a better effect of paying attention to global context information. Inspired by YOLO [12], Chen et al. [13] proposed a lane detection algorithm for end-to-end training and real-time detection. Several horizontal lines with equal spacing are selected in the representation form of the lane lines. Each lane line intersects with these horizontal lines. These intersection points represent the position of the lane line points. Xu et al. [14] proposed a lane-sensitive structure search framework to capture relevant global context information and accurate short-distance lane line curve information so as to solve the difficult problem of curve lane detection in lane line detection. Zheng et al. [15] proposed a crosslayer thinning network to refine the detection results of lane line instances through feature extraction of the features output at different levels of the network. In addition, the IoU loss (Lane IOU—LIoU) based on the line anchors was proposed by referring to the IoU loss function of the anchor frames.

In order to improve the speed and accuracy of lane detection, some research has designed a method of fitting lane lines with function curves according to the prior characteristics of the lane shape, and this work has realized lane instance detection through model prediction function parameters. Van et al. [16] proposed a differentiable least square fitting module for predicting relevant parameters through deep neural networks so as to complete the detection of lane lines. Tabelini et al. [17] proposed a convolutional neural network model that directly predicts polynomial parameters, and the feature graph extracted by the feature network was directly expressed in the form of polynomial parameters through transformation. As the transformer self-attention method has gradually been playing a role in the field of computer vision, Liu et al. [18] built a model feature extraction network based on transformer, and they then fused the extracted feature matrix to directly output polynomial parameters through the fused feature matrix. Feng et al. [19] proposed a parameter fitting method based on the Bessel curve. Considering the particularity of the lane structure, the algorithm flipped and superimposed the feature map in the feature extraction stage to improve the accuracy and anti-interference ability of the lane detection.

The other part of our study uses key point position regression to detect lane lines. This kind of algorithm uses continuous key points to describe lane lines, and it predicts the corresponding key points through a neural network model so as to realize lane line instance detection. Ko et al. [20] proposed a carriage line recognition and detection model based on key points. The feature extraction network of this model is composed of multiple hourglass networks commonly used for key point estimation in series, and the auxiliary training method of knowledge distillation has also been adopted due to its special structure. Qu et al. [21] adopted the encoder–decoder structure as the backbone network structure of the model, in which the decoder network was redesigned for the particularity of lane key point detection. Wang et al. [22] proposed a global association network (GANet) to describe the lane detection problem from a new perspective, in which each lane key point was directly regression calculated with the starting point of the lane line, and a lane perception feature aggregation module was proposed to enhance the correlation between adjacent key points and supplement local information.

The lane detection algorithm based on row classification is similar to the lane detection algorithm based on key points. This method also represents lane instances through a sequence of lane key points. The difference is that this method divides the input image into multiple grids by row, and it uses discontinuous grid classification to determine the exact position of the lane lines in each grid row [23,24]. Yoo et al. [25] used the encoder network as the feature extraction network and then sent the extracted feature map into the detection head network. Different from the literature [23], there was no detection head network designed to assist training, but more confidence detection was carried out for the grid representation of the lane lines. Jayasinghe et al. [26] proposed a lightweight, end-to-end lane detection model based on row classification, which uses special postprocessing technology to achieve efficient and fast lane detection.

### 2.2. Lane Attribute Information Detection

Collado et al. [27] used a lane detection method based on traditional image processing, realized the detection of lane instances through Kalman filter, and used the Fourier analysis method to distinguish them into dotted or solid lines on the basis of the detection of lane instances. Schubert et al. [28] used an example detection method similar to that in the literature [27] and then used a Bayesian classifier to distinguish the lane lines into dotted lines or solid lines. Vpgnet [29] constructed a small traffic marking data set in night and rainy days, divided pixels into various types of lane line categories through semantic segmentation guided by vanishing points, and then realized instance division of the lane line pixels through clustering. Qiao et al. [2], based on the example detection method of lane lines in the literature [18], expanded the branch originally used to detect the existence of lane lines into the category detection branch of lane lines, which can recognize different categories of lane lines but is still limited to the category detection of the overall type. Pizzati et al. [3] added lane attribute information to the TuSimeple data set, but they only added an attribute label to each lane instance, based on which the lane instance and attribute detection method was implemented. Li et al. [4] proposed a multiclass lane semantic segmentation method to realize the detection of different lane categories. Knowledge distillation [30] is a method that can effectively reduce the parameters and depth of the neural network model. Based on this, Hou et al. [5] proposed a new feature extraction method for lane line category detection based on semantic segmentation. First, the given road scene image was decomposed into different regions, and each region was represented as a node in the relational graph. Then, according to the similarity of the nodes in feature distribution, the corresponding relationship between nodes was established so as to form the feature correlation graph between regions, thus effectively transferring the “knowledge” on the scene structure from the teacher model to the student model and improving the performance of the lightweight model.

## 3. Methods

**Figure 1:**
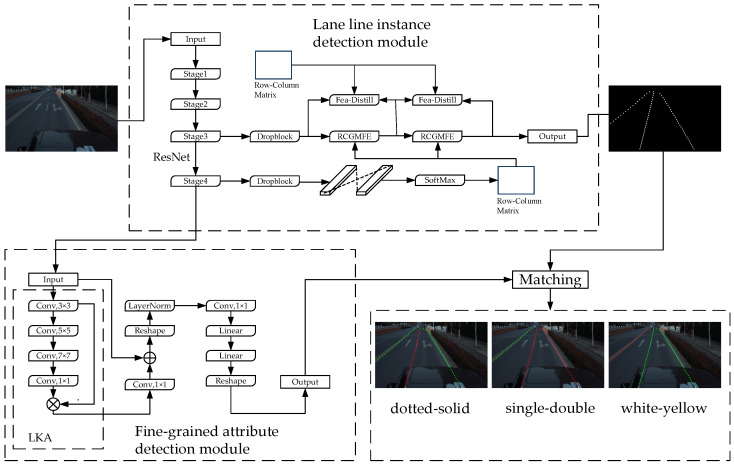
The overall structure of Lane-FGA model.

As shown in Figure 1, this paper decouples the detection tasks of lane line position information and fine-grained attribute information, and it designs a fine-grained attribute detector for dynamic detection of lane line attribute categories. By matching the detection result of the lane line fine-grained attribute with the detection result of the lane line position information, the lane line instance can be detected more completely, which is called the lane line fine-grained attribute detection model.

The first part is the location information detection of lane line instances. Currently, the existing lane detection methods based on deep learning mainly include the lane detection method based on row classification, the lane detection method based on segmentation, the lane detection method based on the line anchor, the lane detection method based on parameter fitting, etc. In this paper, the lane location information detection algorithm based on line classification is designed, and the positive sample grid detection algorithm of lane lines is designed in combination with the target detection idea of anchor frame regression so as to solve the problem of insufficient accuracy caused by the intersection of lane lines with multiple grids in the same row. The row-column guide matrix module is designed to enhance the lane detection ability in complex scenes such as occlusion, as well as improve the recall rate of the model through global information. A feature distillation module, which is sensitive to lane features, is designed to improve the accuracy of lane detection by transmitting lane feature information in different positions of the model.

The second part is the fine-grained attribute detection of lane line instances. The complete attribute distribution of lane lines is described by pixel-level attribute sequence points (descriptors). Aiming at the characteristics of lane fine-grained attribute detection, a lane fine-grained attribute detection module based on global features is designed to ensure the integration of global information and local details through context information and residual connection so as to accurately perform dynamic lane attribute detection.

In addition, although different types of lane location information detection methods have different representation forms of lane instances, they can all be converted into the form of coordinate sequence points through corresponding sampling methods, that is, a complete lane instance is represented by sequence $\begin{array}{c} L=\left\{\left(x_{1},y_{1}\right),\left(x_{2},y_{2}\right)\text{......}\left(x_{n},y_{n}\right)\right\} \end{array}$. Then, by using cubic spline curve, the lane line sequence L is extended to the same dimension as the fine-grained attribute descriptor according to its y value, and new lane line instance detection results are output so as to realize the complete attribute detection of the lane lines.

In Table 1, a comparison between the existing classification methods of lane attributes and the fine-grained attribute detection methods of lane lines in this paper is listed. It can be seen that the lane classification [3] method of lane attribute classification cannot meet the requirements of both lane instance detection and lane fine-grained attribute detection due to the limitations of the model structure and training data.

### 3.1. Lane Line Instance Detection Method

**Figure 2:**
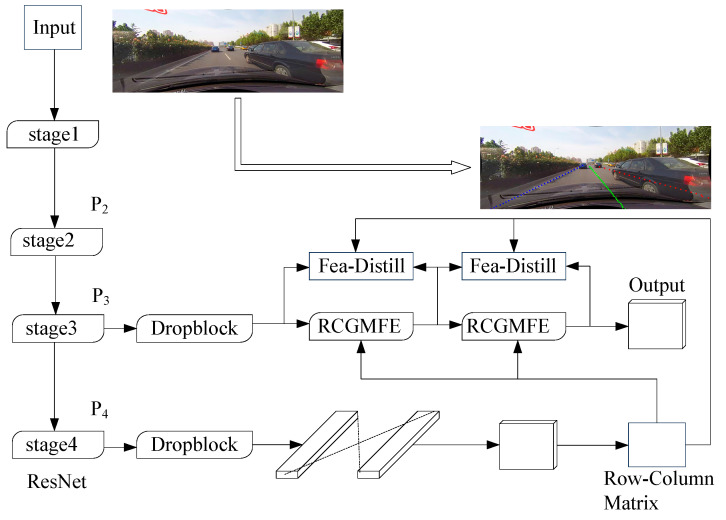
Network structure of lane detection method based on attention guidance.

The algorithm framework of lane position information detection designed in this paper based on the line classification idea is shown in Figure 2. The overall structure takes ResNet as the backbone network, and the Dropblock layer is added to randomly deactivate some neurons to reduce the risk of overfitting of the model. The fourth level feature matrix (P4) output from the model is used to calculate the column guide matrix, and the third level feature matrix (P3) is used to detect the final lane position information. The third level features of the model and the row–column guide matrix participate in the calculation of the feature enhancement module (RCGMFE) to improve the ability of the model to extract the lane features, and the feature distillation module is applied between the feature enhancement modules. The feature distillation module only takes effect during model training and is used to enhance the training effect of the model. Finally, the lane position information is output through the lane position information detector designed in this paper.

#### 3.1.1. Positive Sample Grid Detection of Lane Lines

**Figure 3:**
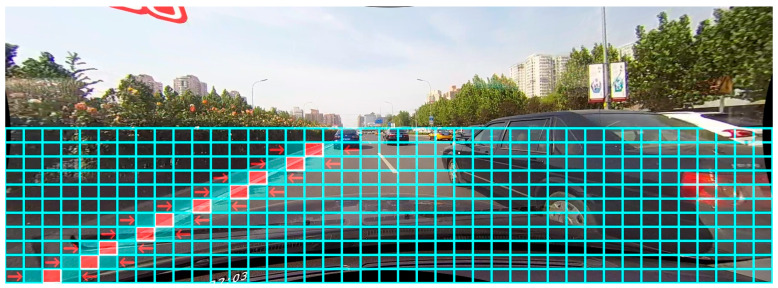
Row classification method network distribution.

As shown in Figure 3, the grid filled with red is the grid through which the center line of the lane lines passes, and the grid filled with other colors represents the multiple grids intersecting with the actual lane lines. Existing row classification lane line detection methods choose to calculate the position expectation in the same row grid as the final lane line position. However, this method has major limitations. The closer the lane line is to the edge of the image, the greater the error will be. One of the reasons is that the grid farther away from the center of the lane line will contribute less to the lane line detection, and on the contrary, it will bring more negative effects due to the participation in expectation calculation.

In view of this, this paper adds the anchor frame-based target detection idea to redesign the lane classification detection method to solve the above problems. All the predetermined grids in the image are regarded as prior anchor frames, and the key points sampled at equal intervals from the center lines of lane lines are regarded as the center points of the true values required in the target detection task. According to the method of target detection, the prior anchor frame is used to detect each sampling point of the lane line by regression. Firstly, the grid distribution is redesigned. Since the artificially designed grid is not only the basis of the lane line detection method of row classification, but it is also the prior anchor frame in the method of this paper, when designing the grid distribution in this paper, the main consideration is to make its width conform to the actual width of the lane lines rather than a large number of presets of row anchor grids, as is done in the traditional row classification method. Secondly, the problem of intersection between lane lines and multiple grids is solved by using the positive and negative sample detection method in object detection. The classification of positive and negative samples is determined by the threshold of intersection, and all the mesh whose IoU with the true value box of lane lines are greater than the threshold is marked as positive sample grids. Finally, when detecting lane lines, the lane line grid belonging to the positive sample in the grid is predicted by the model, and then only the line classification method is used in the positive sample grid to determine the actual grid through which the center line of the lane line passes. For obstacle occlusion, a cubic spline curve is used to mark the true value of the lane line, which is used as the target value of model training so that the prior information is integrated into the target of model training. When reasoning, the equation is as follows:

(1) $$\begin{array}{c} C_{m,n}=\mathrm{argmax}\left(\mathrm{sigmoid}\left(D_{v,m,n}\right)\right) \end{array},v\in \left[1,\;I+1\right],m\in \left[1,\;M\right],n\in \left[1,\;N\right]$$

where I is the maximum number of lane lines detected, M is the preset number of spaces, N is the number of spaces per line, ***C*** is the distribution matrix of the positive sample grid, and $D_{v,m,n}$ is the feature matrix directly output by the model.

Through Equation (1), the matrix output of the model is calculated, that is, the first dimension of matrix ***D***, and the positive sample grid information of the lane line is obtained. Then, on the computed positive sample grid matrix, the grid row direction is classified using Equation (2), and the third dimension of matrix ***D*** is calculated instead of the conventional non-maximum suppression (NMS) calculation. The maximum response position of different lane lines on each grid line is calculated from the positive sample grid matrix, and the detection result of lane lines passing the grid is obtained.

(2) $$\begin{array}{c} L_{i,m}=\left\{\begin{array}{c} \mathrm{argmax}(D_{i,m,n}),C_{m,n}\neq I+1\\ 0,C_{m,n}\equiv I+1 \end{array}\right. \end{array},i\in \left[1,\;I\right],m\in \left[1,\;M\right],n\in \left[1,\;N\right]$$

where $L_{i,m}$ is the positive sample grid with the largest response in the same lane line in the same row.

For each detected lane line prior to the center point coordinates of the anchor frame, follow the border regression adjustment strategy of target detection, but ignore the regression adjustment of the anchor frame size. Finally, the center coordinate of the anchor frame is taken as the position coordinate of the lane line instance, and the equation is as follows:

(3) $$\begin{array}{c} W_{i,m}=\left\{\begin{array}{c} L_{i,m}+O_{m,L_{i,m}},L_{i,m}\neq 0\\ 0,L_{i,m}\equiv 0 \end{array}\right. \end{array},i\in \left[1,\;I\right],m\in \left[1,\;M\right]$$

where $O_{m,L_{i,m}}$ is the value corresponding to the positive sample grid in the offset value matrix O, and $W_{i,m}$ is the detection value of the position of the lane line.

#### 3.1.2. Row–Column Guide Matrix Design

In the urban road environment, lane line occlusion occurs more frequently, and the usual practice is to detect lane line information in complex areas such as occlusion by reasoning through spatial context information. Although the original classification method has the ability of fast lane detection, its structure is too simple, and the detection ability is not enough in the complex urban environment.

In order to improve the accuracy of lane detection in the absence of visual clues such as occlusion, this paper designs an attention matrix calculation method based on the modeling idea of lane detection method based on row classification. Multiple functions related to row classification modeling constrain the feature matrix output of the model so that the final calculated attention matrix is distributed. In line with the distribution law of the foreground and background of the lane line, it is called the row-column guide matrix. Then, the intensity of the lane feature in complex areas such as occlusion is enhanced by the attention matrix.

First, the highest-level feature matrix (P4) with the maximum receptive field is used as the input feature of the row-column guide matrix calculation, and the global context information contained in it is fully utilized. Then, the feature matrix ***Q*** with shape is output through the fully connected neural network layer. Finally, the final column guide matrix A is calculated using Equation (4).

(4) $$\begin{array}{c} A_{m,n}= \end{array}\sum_{i=1}^{I}\mathrm{softmax}\left(Q_{i,m,n}\right)$$

where $A_{m,n}$ is the row-column guide matrix, and ***Q*** is the characteristic matrix of the model output with the shape I×(M+1)×(N+1).

Equation (4) calculates the third dimension of the feature matrix ***Q***, and it follows the way of row classification to calculate the I probability values of each prior anchor box, thus corresponding to I different lane instances. Then, the probability that each prior anchor box is distributed on I lane line instances is accumulated to obtain the distribution law of the foreground and background of lane lines.

In order to make the distribution of matrix $A_{m,n}$ conform to the law of the foreground and background distribution of lane lines, the loss function in the original lane classification detection method is applied to matrix ***Q***, that is, the classification loss function is used in the row direction of matrix ***Q***. At the same time, considering the uneven distribution of the key points in the row direction, the focus loss function is applied to the row direction of the matrix ***Q***, and the first N values of the third dimension of the matrix ***Q*** are ignored during calculation. The loss function is shown in Equation (5).

(5) $$\begin{array}{c} Loss_{\mathrm{r-atten}}=\sum_{i=1}^{I}\sum_{m=1}^{M}L_{\mathrm{CE}}\left({\hat{L}}_{i,m},Q_{i,m,n+1}\right)+\sum_{i=1}^{I}\sum_{m=1}^{M}L_{\mathrm{FL}}\left({\hat{L}}_{i,m},Q_{i,m,n}\right) \end{array}$$

where $Loss_{\mathrm{r-atten}}$ is the loss generated by the constrained attention matrix, ${\hat{L}}_{i,m}$ is the true value of the lane line passing through the grid, $L_{\mathrm{CE}}$ is the crossentropy function, and $L_{\mathrm{FL}}$ is the focus loss function.

**Figure 4:**
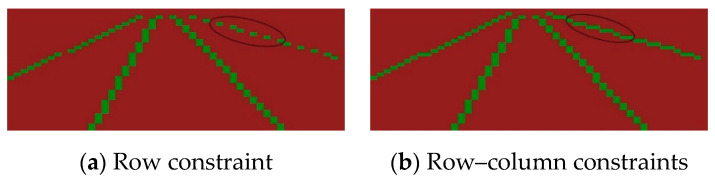
The distribution of true values of the constraints of the guide matrix.

In addition, when the artificially designed grid distribution detects the lane lines located at the edge of the image, the sparsity is more obvious, as shown in Figure 4a, which makes the constraint of the row classification loss function insufficient. The distribution of the true value of the lane lines in different rows is greatly different, and the model output is more prone to errors. In the literature [24], the detection problem of such lane lines is solved through column and row hybrid detection. However, this method will repeatedly detect any lane line, and the selection of repeated detection information is too dependent on the characteristics of the data set. In contrast, this paper applies Equation (5) to the column direction of matrix ***Q*** again to produce constraints in the column direction. The resulting matrix has both the foreground and background distribution law of lane lines with mixed column and row distribution, and it then adds global information into the third level feature (P3) of the model through the row-column guide matrix to guide the model to learn edge sparse features instead of detecting the lane position information directly by using the mixed method of the row and column. The constraint function of the final row-column guide matrix is calculated as follows:

(6) $$\begin{array}{l} \begin{array}{c} \begin{array}{l} Loss_{\mathrm{atten}}=Loss_{\mathrm{r-atten}}+Loss_{\mathrm{c-atten}}\\ =\sum_{i=1}^{I}\sum_{m=1}^{M}L_{\mathrm{CE}}\left({\hat{L}}_{i,m},Q_{i,m,n+1}\right)+\sum_{i=1}^{I}\sum_{m=1}^{M}L_{\mathrm{FL}}\left({\hat{L}}_{i,m},Q_{i,m,n}\right) \end{array} \end{array}\\ +\sum_{i=1}^{I}\sum_{n=1}^{N}L_{\mathrm{CE}}\left({\hat{T}}_{i,n},Q_{i,m+1,n}\right)+\sum_{i=1}^{I}\sum_{n=1}^{N}L_{\mathrm{FL}}\left({\hat{T}}_{i,n},Q_{i,m,n}\right) \end{array}$$

where $Loss_{\mathrm{attn}}$ is the loss generated by the constrained attention matrix, $Loss_{\mathrm{r-atten}}$ is the row constraint of the eigenmatrix of Equation (5), $Loss_{\mathrm{c-atten}}$ is the column constraint of the eigenmatrix, ${\hat{L}}_{i,m}$ is the true value of the lane line passing through each grid row, and ${\hat{T}}_{i,n}$ is the true value of the lane line passing through each grid column.

**Figure 5:**
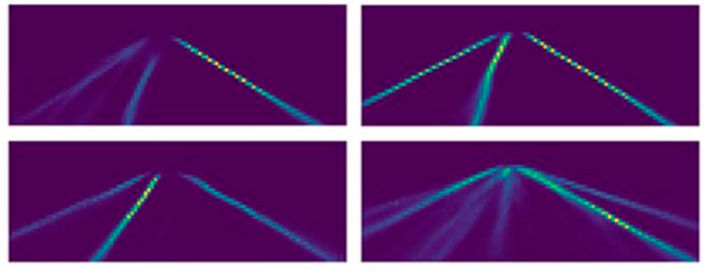
Row–column guide matrix visualization.

Different from other attention methods, the calculation of the attention matrix in this paper relies on the lane detection method of row classification and the detection idea of the positive sample grid, which is called the row-column guide matrix. Its essence is to make the numerical distribution of the matrix output by the model conform to the distribution law of the foreground and background of the lane lines through multiple loss function constraints. The visualization is shown in Figure 5. The bright colored areas in the figure are areas where lane lines may exist.

**Figure 6:**
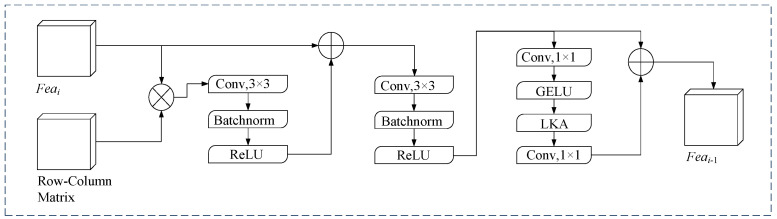
Row–column guide matrix feature enhancement module (RCGMFE) structure diagram.

Based on the row-column guide matrix, this paper designs a feature enhancement module (RCGMFE), which combines the third-level feature matrix output with the backbone network with the row-column guidance matrix to enhance the feature intensity of the model’s interest region and detects the lane position information in complex scenes by using spatial context information. The structure is shown in Figure 6. The row-column guide matrix is multiplied with the third-level feature matrix of the model, and then the boundary between the region and the remaining region is enhanced by fuzzy features through a convolution operation. By connecting with the residual of the original feature matrix, the feature intensity of the noninterest region suppressed by the row-column guide matrix is restored, and the fault tolerance of the model is enhanced.

Inspired by the feature diffusion method in reference [31], this paper introduces the Large Kernel Attention (LKA) module. Large nuclear convolution is an attention module that can effectively enhance the model’s receptor field, and using large nuclear convolution to construct the correlation between different features is an effective method to enhance the model’s performance. However, ordinary large nuclear convolution also has obvious disadvantages, that is, it will bring a lot of calculations and parameters. Due to this limitation, the LKA convolution module solves the above problem by decomposing a large nuclear convolution operation.

#### 3.1.3. Characteristic Distillation Module Design

In deep learning, knowledge distillation is a common method of model compression and training, which is usually used between teacher models and student models. Among them, the performance of the teacher model is better, and the computational complexity of the student model is lower. The teacher model is used to produce constraints on the student model during model training so as to improve the performance of the student model. In the detection model based on a convolutional neural network, the closer to the features extracted by the detection model, the more explicit the semantic features are. Therefore, in the lane position detection network proposed in this paper, a lane feature distillation module is designed, and the feature matrix with significant lane semantic information is taken as the learning target to constrain the output features of the model backbone network.

**Figure 7:**
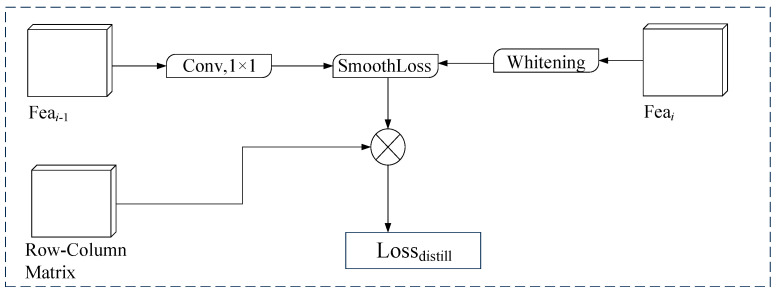
Feature distillation module structure diagram.

Reference [32] studied the effect of feature distillation of the foreground and background information in images on the model, and they proved that the effect of distilling the whole information of images at the same time was even worse than that of distilling only the foreground information or background information; they proposed a feature distillation method with foreground and background differentiation. Meanwhile, the column and row guidance matrix proposed in Section 3.1.2 of this paper essentially generates different guidance weights for the foreground and background information of lane lines. Therefore, a feature distillation module (Fea-Distill) is designed in this paper based on the column and row-column guidance matrix. The structure is shown in Figure 7, and the prefeature matrix and postfeature matrix of the model are input to calculate losses. The pre-eigenmatrix is used to constrain the model.

In the feature distillation module, the feature maps of the features before and after the input of the model are matched in dimension by convolution, that is, the information on the number of channels is matched. The column and row-column guidance matrix is introduced to make the foreground position and the background position in the feature map have different loss weights to avoid the negative impact caused by the simultaneous distillation of the foreground information and the background information so that the feature information of the foreground area of the lane line is mainly transmitted to the backbone network of the model during the distillation process. At the same time, the closer the features are to the output end, the more special the value distribution is. Direct distillation will affect the generalization ability of the model. The whitening process has been introduced in the literature [33] to normalize the characteristics of teachers to reduce redundant information. Therefore, in this paper, the whitening process is applied to the rear feature matrix near the output end, and finally, through the structure design of crosslayer distillation, the feature matrix information of the model is transmitted from back to forward so as to improve the detection performance of the model.

### 3.2. Fine-Grained Attribute Detection Method

#### 3.2.1. Lane Line Fine-Grained Attribute Descriptor

Under normal circumstances, lane lines have six attributes of three types (dotted and solid, single and double, and color). In addition, there are special cases where the curb is used as the lane boundary, and the dotted and solid color attributes of two lane lines are different in the case of dual lanes. Therefore, the classification method of lane line attributes has higher requirements, that is, a more fine-grained dynamic attribute representation method. Most of the previous classification methods of lane attributes cannot be adapted. To solve this problem, this paper sets an additional attribute without visual cues for each type of lane line, and it implements pixel-level fine-grained attribute representation for the same lane line instance. 

In this paper, we choose to decouple three types of attribute distributions to represent the fine-grained attributes of lane lines. Considering that lane lines have long prior characteristics, this paper describes the fine-grained attribute information of the lane lines by setting attribute key points. The dotted and solid attributes, as well as the color and single and double attributes of each attribute key point, are judged, and the fine-grained attribute information of the fixed lane line instance is output.

The fine-grained lane line instance attribute is represented as $T=\left\{\left(sd_{1},wy_{1},ds_{1},\right),\left(sd_{2},wy_{2},ds_{2},\right)\ldots \left(sd_{h},wy_{h},ds_{h}\right)\right\}$. By using dense discontinuous descriptors to represent all the fine-grained attribute distributions of a lane instance, dynamic lane attribute representation is achieved instead of the single-lane classification descriptor of the previous method, where $h\in \left[1,H\right]$, H represents the maximum number of attribute key points of each lane instance; *sd* (single–double) stands for the single–double property; and $sd\in \left[0,3\right]$, 0 indicates that the key point of the attribute is the nonlane line area; 1 indicates the single-line attribute key point, 2 indicates the double-line attribute key point, and 3 represents an additional set of nonvisual cue attribute key points (curbs, among others). The $wy\in \left[0,3\right]$ (white-yellow) color property and $ds\in \left[0,3\right]$ (dotted–solid) dotted line property are set in the same way as the single–double property. Considering that the attributes of the same lane line instance change dynamically in the line type, this means that in order to ensure the continuity of attribute description, H is set with reference to the pixel count of the image in the vertical axis direction.

#### 3.2.2. Fine-Grained Attribute Detection Module

**Figure 8:**
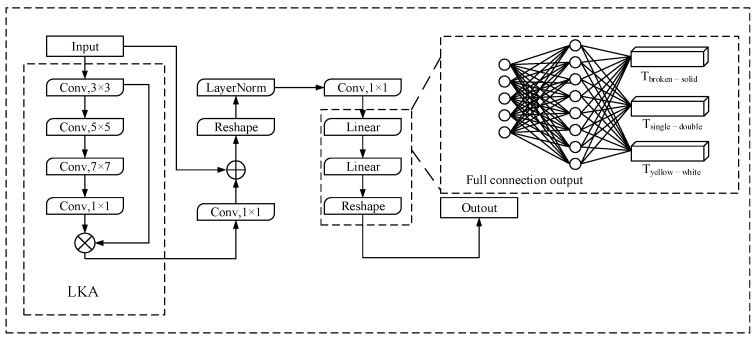
Fine-grained attribute detection module of lane lines.

Different from lane location information detection, lane fine-grained attribute detection has less need to use low-level semantic features for fine detection, and it is more about the segment attribute judgment of lane instances. Especially for the attribute judgment of areas without visual cues such as occlusion, more global context information is needed as the basis of detection. For this reason, the structure of the lane attribute detector designed in this paper is shown in Figure 8.

In Figure 8, the input features are first passed through the LKA, and the convolution characteristics of the LKA are used to enhance the receptive field of the model and obtain more global context information. Then, the residual connection is used to recover the local information of the original feature matrix, and the Layer Norm is used to correct the numerical distribution of the feature matrix to enhance the generalization ability. Finally, the new feature matrix is integrated into the global context information by means of full connection, and the feature matrix is output for each type of attribute. According to Equation (7), the matrix ***T*** output by the model is calculated as the actual fine-grained attribute value of the lane line.

(7) $$\begin{array}{c} P_{i,h}=\max\left(\mathrm{softmax}\left(T_{i,h,k}\right)\right) \end{array},i\in \left[1,\;I\right],h\in \left[1,\;H\right],k\in \left[1,\;K\right]$$

where H is the maximum number of attribute key points of each lane line instance, K is the number of attribute categories of the lane lines, $T_{i,h,k}$ is the weight of prediction of different attributes of the same type of lane line key points, and $P_{i,h}$ is the final attribute value distribution of the same type of lane line key points.

### 3.3. Loss Function

The loss function of this method consists of two parts, namely the loss function of constrained lane position information detection and the loss function of fine-grained lane attribute detection.

Firstly, the loss function $Loss_{cls1}$, which can correctly detect the positive sample information of the lane lines, is calculated by using the crossentropy loss function.

(8) $$Loss_{\mathrm{cls1}}=\sum_{n=1}^{N}\sum_{m=1}^{M}L_{\mathrm{CE}}\left({\hat{D}}_{m,n},D_{v,m,n}\right)$$

where $L_{\mathrm{CE}}$ is the crossentropy function, ${\hat{D}}_{m,n}$ is the true value of the positive sample grid distribution matrix of the lane lines, and $D_{v,m,n}$ is the feature matrix directly output by the model.

Simply applying the idea of target detection to lane detection ignores the prior characteristics of lane lines. Therefore, this paper uses the focus loss function as the row classification loss function to act on matrix ***D***, and it integrates the prior characteristics of the lane lines into the lane detection model through the loss function. The calculation is as follows:

(9) $$\begin{array}{c} Loss_{\mathrm{cls2}}= \end{array}\sum_{i=1}^{I}\sum_{m=1}^{M}L_{\mathrm{FL}}\left({\hat{L}}_{i,m},D_{i,m,n}\right)$$

where $L_{\mathrm{FL}}$ is the focus loss function, $D_{i,m,n}$ is the matrix value of the first I channels in the first dimension of matrix D, and ${\hat{L}}_{i,m}$ is the true value of the lane line passing through the grid.

In this paper, the positive sample of each grid line is determined according to the IoU threshold between the prior anchor frame and the key point target frame of the lane line, and the regression loss from the positive sample grid to the exact position of the lane line is calculated. The regression matrix corresponding to the output is predicted by smoothing the L1 loss function constraint model. The calculation is as follows:

(10) $$\begin{array}{c} {Loss}_{\mathrm{reg}}= \end{array}\sum_{m=1}^{M}\sum_{n=1}^{N}\left\{\begin{array}{c} L_{1}\left({\hat{O}}_{m,n},O_{m,n}\right),{\hat{D}}_{m,n}\neq I+1\\ 0,{\hat{D}}_{m,n}=I+1 \end{array}\right.$$

where ${Loss}_{reg}$ is the prediction loss of the offset value of the positive sample grid of the lane lines, and it is the set of the true values of the positive samples for each row; $L_{1}$ is the smooth L1 loss function; $O_{m,n}$ is the subset of the matrix representing the prediction of the offset value of the accurate position of the positive sample grid and the lane lines; and ${\hat{O}}_{m,n}$ is the offset value of the accurate position of the positive sample grid and the lane line.

The characteristic distillation loss function is a smooth L1 loss function, and the loss values at different positions of the characteristic matrix are multiplied by corresponding weight coefficients during calculation so that the distillation characteristics of the foreground background of the lane lines are fully taken into account during the characteristic distillation. The specific loss function is as follows:

(11) $$\begin{array}{c} {Loss}_{\mathrm{distill}}= \end{array}\sum_{c=1}^{C}\sum_{m=1}^{M}\sum_{n=1}^{N}\left(L_{1}\left({\hat{F}}_{c,m,n},F_{c,m,n}\right)\times A_{m,n}\right)$$

where ${\hat{F}}_{c,m,n}$ is the value of the rear feature matrix near the output end of the model, $F_{c,m,n}$ is the value of the front feature matrix near the backbone network, C is the number of feature matrix channels involved in feature distillation, $A_{m,n}$ is the column guide matrix, and $\;L_{1}$ is the smooth L1 loss function.

In addition, the corresponding constraint function ${Loss}_{\mathrm{attn}}$ has been proposed in Section 3.1.2, which makes the numerical distribution of the row–column guidance matrix output by the model conform to the foreground and background distribution law of the lane lines.

For the fine-grained attribute detection of lane lines, a crossentropy loss function constraint is adopted, and the calculation is as follows:

(12) $$\begin{array}{c} {Loss}_{\mathrm{prop}}= \end{array}\sum_{i=1}^{I}\sum_{h=1}^{H}L_{\mathrm{CE}}\left({\hat{T}}_{i,h},T_{i,h,k}\right)$$

where ${Loss}_{prop}$ is the detection loss of the lane segment attribute, and ${\hat{T}}_{i,h}$ is the true value of the lane segment attribute.

The complete loss function is as follows:

(13) $$\begin{array}{c} {Loss}_{\mathrm{sum}}=\alpha \times {Loss}_{\mathrm{cls1}}+\beta \times {Loss}_{\mathrm{cls2}}+\varepsilon \times {Loss}_{\mathrm{atten}}+\lambda \times {Loss}_{\mathrm{reg}}+\delta \times {Loss}_{\mathrm{distill}}+\theta \times {Loss}_{\mathrm{prop}} \end{array}$$

where ${Loss}_{\mathrm{sum}}$ is the total loss of the lane line complete information detection method in this paper, and α, β, ε, λ, δ, and θ are the weight coefficients of each loss value, which matche the order of magnitude of each loss value.

## 4. Experiments

### 4.1. Construction of Fine-Grained Attribute Data Set of Lane Lines

#### 4.1.1. Lane Line Data Set

CULane is a large open-source data set commonly used in lane detection algorithm research. This paper chose CULane to verify the lane location information detection algorithm in Section 3.1 of this paper. The dataset covers a variety of traffic scenarios, including Normal, crowded, Dazzle, Shadow, Noline, Arrow, Curve, Crossroad, and Night. For each image, the position information of the lane lines is manually marked by cubic splines, and even in complex cases such as when the lane lines are blocked by vehicles, it is also accurately marked according to the context. 

The current open-source data sets are relatively lacking in lane attribute labeling, and most of the open-source lane attribute data sets only label the lane location information at the instance level. The existing open-source lane attribute data set is only annotated with a one-hot code for each lane instance, such as the improved TuSimple dataset [26]. This annotation method is too simple and cannot fully reflect the dynamic changes of lane attributes, such as at intersections, roadside entrances and exits, and other attribute variation areas. Therefore, in order to meet the needs of fine-grained attribute detection of lane lines in urban environment, the detection can reflect the attributes of the lane lines in real time. This section constructs a lane data set with both lane instance information and fine-grained attribute information, which serves as the data basis for the experiment in this paper. The fine-grained attribute data set of lane lines constructed in this section should meet two requirements:

(1) With instance level lane line annotation information (pixel annotation or key point annotation can be possible). For lane lines with no visual clues, such as an occlusion and a dashed line interval, the lane line examples are marked according to a cubic spline curve and other methods.

(2) Each pixel belonging to the lane line in the image should have the corresponding attribute annotation information, including the lane line pixel in the area without visual clues such as occlusions and dashed line intervals.

**Figure 9:**
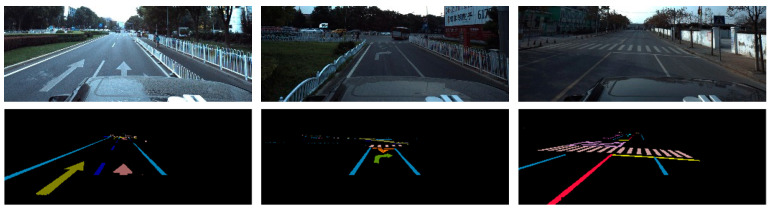
Apolloscape data set.

According to Table 2, for lane instance detection and fine-grained attribute detection, the annotation information of the current open-source lane data set can only meet part of the requirements. Therefore, considering practical application, this paper chose the ApolloScape [34] data set as the basis to build the required fine-grained attribute data set of lane lines. Among them, ApolloScape is a large automatic driving data set with a resolution of 2710 × 3384, including 3D and 2D vehicles, road traffic marking, and other annotation information. Ground marking includes 35 common marking categories and background, pavement damage marking, thus constituting a total of 37 categories. Each class is defined by its type, color, and other attributes, as visualized in Figure 9.

As can be seen from Figure 9, the ApolloScape data set only annotates lane lines with multiple attribute types in the form of semantic segmentation, does not have carrier-level lane line annotation information, and does not meet the requirement (1) of being a fine-grained lane line attribute data set. As for requirement (2), it does not meet the requirement of lane line pixel attribute annotation in complex situations such as occlusion. In addition, commonly used open-source lane data sets, such as CULane, are more difficult to meet the needs of this study and subsequent experiments.

#### 4.1.2. Annotation of Lane Instance Information

**Figure 10:**
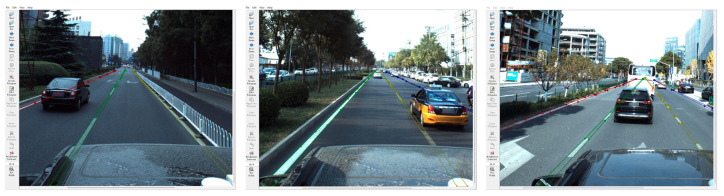
Lane line instance information annotation.

The Labelme annotation tool was used in this paper to annotate the instance-level lane information to supplement the missing lane instance information in the ApolloScape data set, as shown in Figure 10.

In Figure 10, lane line instances in complex areas such as occlusion are marked by context clues, and each lane line instance is completely marked until the far end is invisible. Finally, instance-level information is marked by describing the external outline of the lane line. A total of 5264 instance-level lane samples, called the ApolloScape-Precise data set, were manually labeled in this paper, which were divided into 4160 training samples, 520 test samples, and 584 verification samples.

The manual construction of data sets, while extremely accurate, is too much work. Therefore, this paper constructed an intelligent lane instance labeling process based on an ApolloScape-Precise manual labeling data set and built a labeling model based on the aforementioned lane classification detection methods, split lane detection methods, and anchored lane line detection methods. The backbone network was replaced with ResNet-101 from ResNet-18, and then the annotated model was pretrained using ApolloScape-Precise using CULane as the pretrained data set for the annotated model. The fine-tuned models were used to label the key point sequence of lane instances, generate lane label information, and complete crossverification. Finally, the marked information with obvious errors was removed by manual correction and screening. In the end, a total of 56,828 annotated images were generated in the annotated data set, which was divided into 39,779 in the training set, 11,365 in the test set, and 5684 in the verification set, which was called the ApolloScape-Large data set.

**Figure 11:**
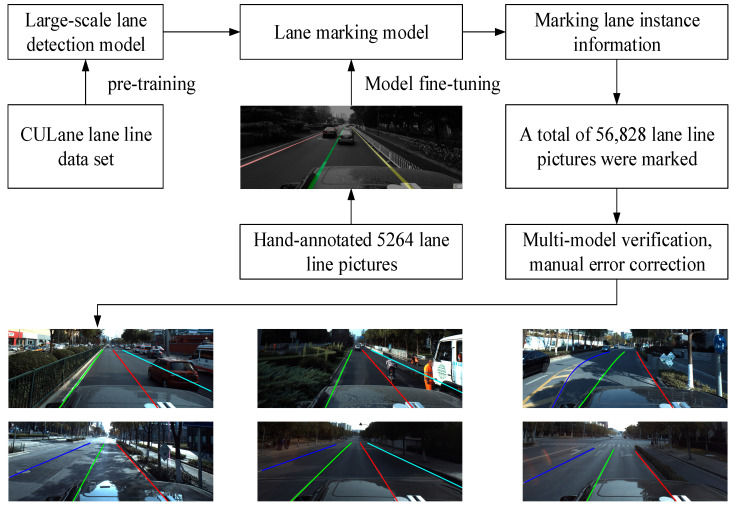
Intelligent lane line instance labeling process.

The results of intelligent lane line annotation in this paper are shown in Figure 11. The advantage of intelligent annotation is that it can greatly reduce the labor cost and time cost, quickly and accurately build a large number of samples, and provide a rich data basis for relevant experiments.

#### 4.1.3. Lane Attribute Completion in Complex Environment

In addition to the lane attribute annotation information of ApolloScape’s original data set, it is difficult to directly annotate the fine-grained attributes of lane lines in complex cases such as occlusion, which need to be inferred based on contextual information. Therefore, this paper designed the attribute reasoning algorithm using ApolloScape’s original annotation information and lane line instance annotation information in Section 4.1.2 as input to iteratively deduce the complete lane line fine-grained attribute information. The fine-grained attributes of the lane lines contain a white-yellow color attribute, a single–double attribute, and a dotted–solid attribute. The following lane line attribute reasoning algorithm takes the dotted–solid attribute as an example, which is suitable for the white-yellow color attribute and single–double attribute.

The inference process of each lane line instance is shown in Algorithm 1, where the element value of input matrix **Q** is 1 or 0, where 1 and 0 respectively represent whether the attribute point is a lane line area or not. The values of elements in the input matrix **D** are, respectively, 0, 1, 2, and 3, where 0 indicates that the attribute point is a nonlane line area, 1 indicates that the attribute point has no visual cue, 2 indicates that the attribute point is a dotted line, and 3 indicates that the attribute point is a solid line.
**Algorithm 1:** Lane line attribute inference in areas without visual cues**Input: D**, **Q**, **P**, where **D** is the visual attribute label matrix; **Q** is the distribution matrix of lane instances; **P** is a complete fine-grained attribute matrix.**Initialization:** Matrix **Q** is initialized according to the lane instance annotation information in Section 4.1.2. Matrix **D** is initialized according to the original annotation information of ApolloScape, and the missing region is set to 0; **P** = **D**; h is equal to 2.1   **Repeat**2     **if D***_h_* = 0 **and Q***_h_* > 0 then 3      **if D***_h_*_−1_ > 0 **then** 4        **P***_h_* = **D***_h_*_−1_    /* Inferring nonvisual region attribute values from the bottom up */ 5      **else** 6        **P***_h_* = 1              /* Judged as special areas such as curbs */ 7      **end if** 8     **end if**9     *h* = *h* + 1                   /* Upward iterative computation */10  **Until** *h* = H 11  *h* = H − 1 12  **Repeat** 13     **if P***_h_* = 1 **and P***_h+_*_1_ ≠ 0 **then** 14      **P***_h_* = **P***_h+_*_1_  /* Reasoning from top to bottom The region cannot be determined in Step 6 */ 15     **end if** 16     *h* = *h* − 1                 /* Downward iterative computation */ 17  **Until** *h* = 0**Output**: **P**

The detailed steps are as follows:

a. Select the second attribute key point near the bottom of the image to start;

b. Use the visual label as a clue; if the key point is in the solid line marking area, then set the attribute of the point to the solid line; if the key point is in the dotted line marking area, the property of the point is set to a dotted line;

c. If the key point is neither in the solid line area nor the dotted line area but belongs to the lane line marking range, it is judged according to the attribute of the previous key point. If the previous attribute detection point belongs to the lane line area, the attribute detection point label is set to be the same as the previous attribute detection point; otherwise, it is set to be a no clue label;

d. If the key point does not belong to the range of lane markings, ignore it;

e. Select the next attribute point and repeat b–e to complete the subsequent key point attribute calculation;

f. Select the top of the image, that is, the second attribute key point at the far end;

g. If the key point is a clueless label, perform the following steps; otherwise, select the next attribute detection point and repeat step f;

h. If the previous attribute detection point is not a nonlane line label, the attribute detection point label setting is the same; otherwise, keep the clueless label value unchanged;

i. Select the next attribute point and repeat g–i to complete the subsequent key point attribute calculation.

**Figure 12:**
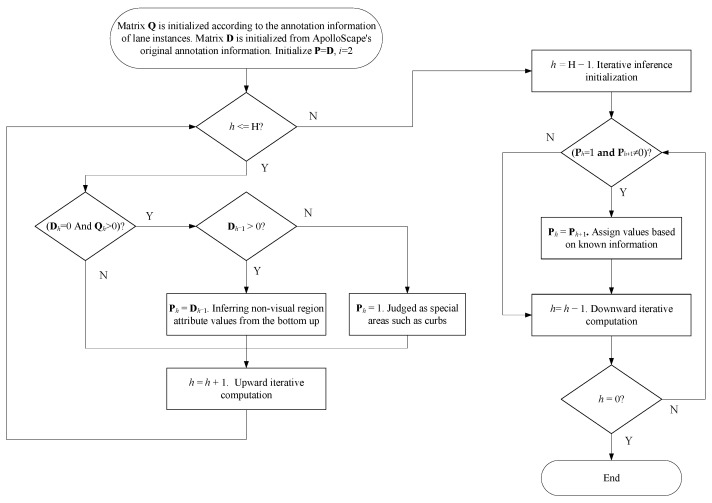
Flow chart of lane attribute inference in areas without visual cues.

**Figure 13:**
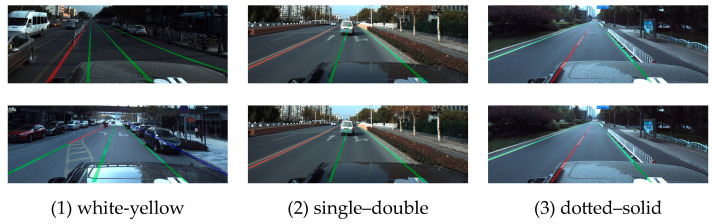
Loop iteration lane line fine-grained attribute inference.

The flow of the lane attribute iterative reasoning algorithm is shown in Figure 12. The algorithm aims to make full use of the context information in the image. When only the top-down or bottom-up reasoning flow is used, the dependent information only comes from one side of the attribute point, and when the unilateral side is the lane line area without visual cues, the basic information that can be relied on will be lacking, thus resulting in the loss of part of the value of the attribute reasoning matrix **P**. In contrast, cyclic iterative reasoning temporarily ignores the current attribute point when there is no basic information for the first time, and it relies on the information at the top of the image for the second time. The visual result of attribute completion is shown in Figure 13.

The specific information of the data set used in this experiment is shown in Table 3.

### 4.2. Evaluation Index and Experimental Setting

#### 4.2.1. Lane Detection Evaluation Index

For lane line location information detection, this paper used the comprehensive evaluation index F1 to measure the detection performance of the model. The precision rate is the ratio of the number of correctly detected lane instances TP to the total number of detected lane instances TP + FP (the sum of the number of correctly detected and incorrectly detected instances). The recall rate is the ratio of the number of correct lane line instances detected to the number of lane line instances TP+FN of all marked true values. The comprehensive evaluation index F1 is calculated from the precision rate and the recall rate.

(14) $$\begin{array}{c} \mathrm{Precision}=\frac{\mathrm{TP}}{\mathrm{TP}+\mathrm{FP}} \end{array}$$

(15) $$\begin{array}{c} \mathrm{Recall}=\frac{\mathrm{TP}}{\mathrm{TP}+\mathrm{FN}} \end{array}$$

(16) $$\begin{array}{c} F1=\frac{2\times \mathrm{Precision}\times \mathrm{Recall}}{\mathrm{Precision}+\mathrm{Recall}} \end{array}$$

For the fine-grained attribute detection of lane lines, the accuracy rate is used as an evaluation index, and each attribute category is evaluated separately. Take the dotted and solid attribute as an example, including the accuracy of identifying the key point of the dotted attribute, the accuracy of identifying the key point of the solid attribute, and the average accuracy. The evaluation indexes of singled–double and white-yellow attributes are set the same, and the average accuracy of the three attribute types are called Acc_ds_, Acc_sd_, and Acc_wy_, respectively.

(17) $$\begin{array}{c} {\mathrm{Acc}}_{\mathrm{dotted}}=\frac{\sum_{\mathrm{dotted}}C_{\mathrm{dotted}}}{\sum_{\mathrm{dotted}}S_{\mathrm{dotted}}} \end{array}$$

(18) $$\begin{array}{c} {\mathrm{Acc}}_{\mathrm{solid}}=\frac{\sum_{\mathrm{solid}}C_{\mathrm{solid}}}{\sum_{\mathrm{solid}}S_{\mathrm{solid}}} \end{array}$$

(19) $$\begin{array}{c} {\mathrm{Acc}}_{\mathrm{mean}}={\mathrm{Acc}}_{\mathrm{dotted}}\frac{S_{\mathrm{dotted}}}{S_{\mathrm{dotted}}+S_{\mathrm{solid}}}+{\mathrm{Acc}}_{\mathrm{solid}}\frac{S_{\mathrm{solid}}}{S_{\mathrm{dotted}}+S_{\mathrm{solid}}} \end{array}$$

where $C_{\mathrm{dotted}}$ is the key point for detecting the correct dotted line attribute, $S_{\mathrm{dotted}}$ is the key point for marking the dotted line attribute, $C_{\mathrm{solid}}$ is the key point for detecting the correct solid line attribute, and $S_{\mathrm{solid}}$ is the key point for marking the correct solid line attribute.

#### 4.2.2. Specific Parameters

The experimental environment built in this paper is based on Ubuntu 20.04 as the operating system, PyTorch 1.8 as the deep learning framework, and Python 3.8 as the main development language. In terms of computing devices, the GeForce RTX 3090 was selected.

In the training parameter setting of the experimental model, Adam was selected as the optimizer, and the initial learning rate was set to 1 × 10^-4^ At the same time, CosineAnnealingLR was used as the learning rate decline function, and the positive sample threshold was set to 0.25. In the training process of the CULane data set, a total of 36 row anchors were set, and each row was evenly divided into 50 grids. A total of 15 epochs were carried out in the whole training process, and each small batch contained 24 picture information. A total of 36 row anchors were set on the ApolloScape-Precise data set. Each row was evenly divided into 100 grids, and 100 epochs were trained. Each small batch contained 8 picture information. A total of 36 row anchors were set on the ApolloScape-Large data set, each row was evenly divided into 100 grids, and a total of 30 epochs were trained. Each small batch contained 12 picture information, and 106 attribute keys were set for each lane instance.

### 4.3. Comparison Experiment of Lane Position Information Detection

In this section, segmentation-based lane detection methods, anchor-based detection methods, key point-based lane detection methods and row and column mixed classification lane detection methods have been selected for comparison.

Table 4 shows the detection results of the proposed method and various types of mainstream lane detection methods on the CULane data set. For the fairness of algorithm comparison, the residual network ResNet was selected as the backbone network, which can be replaced in practical application, and the index calculation parameters of each method were the same. In this paper, the anchor frame-based target detection method was first applied to lane detection, and then the traditional NMS was replaced with the lane detection method of row classification. Finally, the attention guide matrix and feature distillation method were designed to effectively improve the accuracy of lane detection. In many scenarios, the F1 index of the proposed method was better than that of the original classification lane detection method.

Compared to various types of lane detection methods, the proposed method has advantages in various types of lane detection methods. Under the ResNet-18 backbone network, the comprehensive evaluation index F1 in conventional driving scenarios reached 92.8%, thus indicating that the method has extremely high detection accuracy in normal driving environments. In complex driving scenarios such as congestion, glare, shadow, and night, although the detection difficulty increased, the proposed method could still maintain relatively stable performance, with F1 values reaching 76.0%, 71.8%, 77.0%, and 73.3%, respectively. These results fully demonstrate the effectiveness and robustness of the proposed method in a variety of driving scenarios. Furthermore, in order to improve the detection ability of the model in a specific scenario, we considered the problem of uneven data distribution among various scenarios in the CULane dataset. To this end, we pretrained the model on the Curvelanes data set, borrowed the idea of transfer learning, and conducted the training test on the CULane data set based on the weight of the pretrained model. This strategy effectively alleviated the challenge caused by the unbalanced data distribution and further improved the detection performance of the model. Finally, on the ResNet-18 and ResNet-34 backbone networks, the lane evaluation index F1 reached 77.9% and 78.3%, respectively, which not only verifies the effectiveness of transfer learning in lane detection tasks but also once again proves the competitiveness of the proposed method in various types of lane detection methods.

### 4.4. Fine-Grained Attribute Detection Experiment

The fine-grained attribute detection method presented in this paper can be combined with the lane detection method with case detection capability to build a fine-grained attribute detection model for lane lines. In this section, based on the lane detection method based on the column guidance in Section 3.1, the output feature matrix was changed from P3 to P2 to adapt to the image acquisition characteristics of the ApolloScape data set. In order to more accurately demonstrate the detection capability of lane position information and fine-grained attribute information, ApolloScape-Precise was selected as the training and verification data in this section.

The fine-grained attribute detection results of the lane lines on the manually labeled data set ApolloScape-Precise are shown in Table 5. In this section, the residual networks ResNet-18 and ResNet-34 were used as feature extraction networks. The detection accuracy of multiple attribute categories reached more than 85%. As the fine-grained attributes of lane lines have practical significance such as right-of-way information in urban traffic control, it is difficult to avoid the long-tail effect of their data distribution, so the detection accuracy is different among different attributes of the same type. In this paper, a variety of data enhancement methods were adopted to reduce the impact of uneven data distribution.

### 4.5. Comparison Experiment of Fine-Grained Attribute Detection

In addition, in order to prove the compatibility of the fine-grained attribute detection method for lane lines in this paper with various types of lane line detection methods, the lane line detection method based on row classification, the lane line detection method based on segmentation, and the lane anchor detection method were selected for experimental verification in this section.

The results of lane detection based on segmentation are a lane segmentation graph and a pixel category probability graph generated by softmax function. According to the ordinate of the attribute points generated by the fine-grained attribute detection branch, the corresponding one was generated by sampling on the Y axis of the probability graph so as to realize the matching between the location information of the lane line instance and the fine-grained attribute information.

The lane detection method based on line anchor adds a lot of prior design because of its intensive detection characteristics, and it is difficult to directly match the key points of lane position information with the fine-grained attribute key points like the above method. In order to realize the complete attribute detection method of lane lines based on the wire anchor, one method is to modify the original lane line method based on the wire anchor. For any lane line, the preset anchor predicts N + 1 scores, where N is the maximum number of lane lines detected, and the NMS postprocessing method in the original method is removed. Another method is to predict a set of fine-grained attribute key point sequences for each anchor and then match the predicted values of the attribute key point sequences with the real values according to the original matching strategy so as to realize the complete attribute detection of the lane lines. However, this method will bring a lot of unnecessary calculation, that is, the calculation cost caused by the attribute prediction of the negative sample anchor. Therefore, this section chooses the idea of the first method to realize the complete attribute detection method of lane lines based on line anchors.

In the multimethod comparison experiment considering the differences in training strategies and model convergence difficulty of different types of lanne detection methods, as well as the additional segmentation and decoding requirements of other methods for supplementary training, this section generates the true value mask map of the lane lines with a width of 30 pixels based on the key point annotation information of ApolloScape-Large.

The test results of this experiment on the ApolloScape-Large data set generated by intelligent markup are shown in Table 6. The methods in the table all used ResNet-18 as the main backbone network. Among them, the average accuracy Acc_ds_ corresponding to dotted and solid attribute points was more than 97%, the average accuracy Acc_wy_ corresponding to color attribute points was also more than 97%, and the average accuracy Acc_sd_ corresponding to single and double attribute points was about 97%. In addition, the F1 index of lane position information detection and evaluation reached 95%. The experimental results show that the fine-grained attribute detection method in this paper has good accuracy and compatibility, and it can be combined with different types of lane detection methods to achieve more perfect lane information detection.

**Figure 14:**
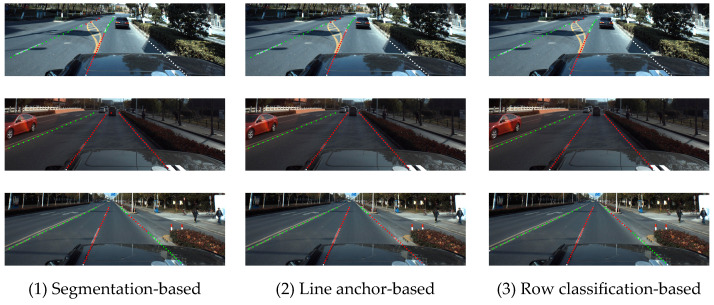
ApolloScape-Large visualization of fine-grained attribute detection results for multitype detection methods.

Figure 14 shows the visualization of the detection results of the white-yellow attribute, single–double attribute, dotted–solid attribute from top to bottom, as well as the fine-grained attribute detection results of various types of lane detection methods added in this section from left to right. Among them, the fine-grained attribute detection method of the lane lines based on row classification designed in this paper had more accurate dynamic detection ability at the critical point of lane instance attribute change, while the fine-grained attribute detection method based on the line anchor was more prone to errors at the critical point. From the overall visualization results, it can be seen that the fine-grained lane attribute detection method designed in this paper can accurately realize the dynamic detection of lane attribute in an urban environment after combining with a variety of different types of lane instance detection methods.

## 5. Conclusions

In order to meet the demand for the more complete information detection of lane lines in an urban environment, this paper analyzed the shortcomings of the current main lane attribute classification methods and proposed a fast and accurate lane detection network, which changed the previous lane attribute detection methods, realized more fine-grained lane attribute detection, and could accurately detect the dynamic changes of the lane attributes. Additionally, it can be compatible with many mainstream lane detection methods. Secondly, on the basis of the ApolloScape data set, the fine-grained attribute data sets of lane lines of Apolloscape-Precise and Apolloscape-Large were constructed by combining manual annotation and intelligent annotation. An attribute inference algorithm based on loop iteration was designed to solve the problem of lane attribute labeling in areas without visual cues. Finally, the effectiveness of the proposed method was fully verified by experiments, and its compatibility with many types of lane detection methods has been demonstrated. The experimental results show that the proposed method can accurately detect the changes of lane alignment in complex areas in an urban environment.

Furthermore, we will build a more complete road traffic marking detection framework, thus aiming to make better use of existing lane detection and other deep learning methods. At the same time, combined with road sign detection, it can realize the task of right-of-way information perception in urban environments more accurately.

## Figures and Tables

**Table 1:** Comparison between lane line attribute classification methods.

Method	Instance	Attribute Classification	Fine-Grained Attribute
Lane-cascaded [2]	√	√	—
Lane classification [3]	√	√	—
Lane DeepLab [4]	—	—	√
Lane-FGA	√	√	√

**Table 2:** Comparison between annotation information in some open-source data sets.

Data Set	Lane Instance Annotation	Fine-Grained Attribute Annotation
CULane	√	—
TuSimple	√	—
ApolloScape	—	* √

Mark * indicates that some requirements are met, but the fine-grained attribute marks of lane lines in blank areas such as occlusion and dotted line interval are missing.

**Table 3:** Data set for experiment analysis.

	Training	Test	Validation	Resolution	Lines
CULane	88,880	34,680	9675	1640 × 590	≤4
ApolloScape-Precise	4160	520	584	3384 × 2710	≤4
ApolloScape-Large	39,779	11,365	5684	3384 × 2710	≤4

**Table 4:** Comparison of F1 index of lane position information on CULane test set.

Type	Method	Normal	Crowded	Dazzle	Shadow	Noline	Arrow	Curve	Crossroad	Night	Total
Segmentation-based	RESA(ResNet-34) [35]	91.9	72.4	66.5	72.0	46.3	88.1	68.6	1896	69.8	74.5
	RESA(ResNet-50) [35]	92.1	73.1	69.2	72.8	47.7	88.3	70.3	1503	69.9	75.3
Curve-based	Bezier(ResNet-18) [19]	90.2	71.6	62.5	70.9	45.3	84.1	59.0	996	68.7	73.7
	Bezier(ResNet-34) [19]	91.6	73.2	69.2	76.7	48.1	87.2	62.5	888	69.9	75.6
Point detection-based	CurveLanes(M) [14]	90.2	70.5	65.9	69.3	48.8	85.7	67.5	2359	68.2	73.5
	CurveLanes(L) [14]	90.7	72.3	67.7	70.1	49.4	85.8	68.4	1746	68.9	74.8
Line anchor-based	LaneATT(ResNet-18) [11]	91.1	72.9	65.7	70.9	48.3	85.4	63.3	1170	68.9	75.1
	LaneATT(ResNet-34) [11]	92.1	75.0	66.4	78.1	49.3	88.3	67.7	1330	70.7	76.6
	O2SFr(ResNet-18) [36]	91.9	73.9	70.4	74.8	49.8	86.1	68.7	2361	70.7	76.1
	O2SFr(ResNet-34) [36]	92.5	75.3	70.9	77.7	51.0	87.6	68.1	2749	72.9	77
Row classification- based	UFLDv2(ResNet-18) [24]	91.8	73.3	65.3	75.1	49.2	87.9	68.5	2075	70.7	75
	UFLDv2(ResNet-34) [24]	92.5	74.8	65.5	75.5	46.8	88.8	70.1	1910	70.8	76
	Lane-FGA(ResNet-18)	92.8	76.0	71.8	77.0	51.2	87.9	73.0	1622	73.3	77.7
	Lane-FGA(ResNet-18) *	92.4	76.5	71.8	78.8	51.6	88.3	72.1	1639	73.7	77.9
	Lane-FGA(ResNet-34) *	93.1	76.9	73.0	77.2	51.7	88.9	72.2	1617	74.4	78.3

* Pretrain for loading Curvelanes.

**Table 5:** ApolloScape-Precise fine-grained attribute detection results.

Attribute	Index	Lane-FGA(ResNet-18)	Lane-FGA(ResNet-34)
Dotted–solid	Acc_dotted_	89.46	90.37
	Acc_solid_	89.01	89.26
	Acc_mean_	89.19	89.71
Yellow-white	Acc_yellow_	93.08	93.37
	Acc_white_	92.62	93.12
	Acc_mean_	92.69	93.16
single−double	Acc_single_	93.43	93.95
	Acc_double_	85.66	88.51
	Acc_mean_	93.19	93.78

**Table 6:** Fine-grained attribute detection index on ApolloScape-Large test set.

Experiment	Type	Acc_ds_	Acc_wy_	Acc_sd_	F1
Experiment 1	Segmentation-based [35]	98.49	97.73	97.57	95.58
Experiment 2	Line anchor-based [15]	98.59	97.79	96.97	96.30
Experiment 3	Row classification-based	97.02	97.87	98.08	96.64

## Data Availability

The data sets generated for this study are available upon request from the corresponding author.
